# Multicenter validation study for automated left ventricular ejection fraction assessment using a handheld ultrasound with artificial intelligence

**DOI:** 10.1038/s41598-024-65557-5

**Published:** 2024-07-04

**Authors:** Nobuyuki Kagiyama, Yukio Abe, Kenya Kusunose, Nahoko Kato, Tomohiro Kaneko, Azusa Murata, Mitsuhiko Ota, Kentaro Shibayama, Masaki Izumo, Hiroyuki Watanabe

**Affiliations:** 1 Department of Cardiovascular Biology and Medicine, Juntendo University Graduate School of Medicine, 2-1-1 Hongo, Tokyo, 113-0021 Japan; 2 Department of Digital Health and Telemedicine R&D, Juntendo University, Tokyo, Japan; 3 Department of Cardiology, Osaka City General Hospital, Osaka, Japan; 4 Department of Cardiovascular Medicine, Nephrology, and Neurology, University of the Ryukyus, Okinawa, Japan; 5 Department of Cardiology, Tokyo Bay Urayasu Ichikawa Medical Center, Urayasu, Japan; 6 Department of Cardiovascular Center, Toranomon Hospital, Tokyo, Japan; 7 Department of Cardiovascular Medicine, Tokyo Cardiovascular and Internal Medicine Clinic, Tokyo, Japan; 8 Division of Cardiology, Department of Internal Medicine, St. Marianna University School of Medicine, Kawasaki, Japan

**Keywords:** Artificial intelligence, Point-of-care ultrasound, Echocardiography, Handheld ultrasound device, Cardiology, Diagnosis, Medical imaging

## Abstract

We sought to validate the ability of a novel handheld ultrasound device with an artificial intelligence program (AI-POCUS) that automatically assesses left ventricular ejection fraction (LVEF). AI-POCUS was used to prospectively scan 200 patients in two Japanese hospitals. Automatic LVEF by AI-POCUS was compared to the standard biplane disk method using high-end ultrasound machines. After excluding 18 patients due to infeasible images for AI-POCUS, 182 patients (63 ± 15 years old, 21% female) were analyzed. The intraclass correlation coefficient (ICC) between the LVEF by AI-POCUS and the standard methods was good (0.81, p < 0.001) without clinically meaningful systematic bias (mean bias -1.5%, p = 0.008, limits of agreement ± 15.0%). Reduced LVEF < 50% was detected with a sensitivity of 85% (95% confidence interval 76%–91%) and specificity of 81% (71%–89%). Although the correlations between LV volumes by standard-echo and those by AI-POCUS were good (ICC > 0.80), AI-POCUS tended to underestimate LV volumes for larger LV (overall bias 42.1 mL for end-diastolic volume). These trends were mitigated with a newer version of the software tuned using increased data involving larger LVs, showing similar correlations (ICC > 0.85). In this real-world multicenter study, AI-POCUS showed accurate LVEF assessment, but careful attention might be necessary for volume assessment. The newer version, trained with larger and more heterogeneous data, demonstrated improved performance, underscoring the importance of big data accumulation in the field.

## Introduction

Technological advancements in recent years have facilitated the development of portable computing devices and miniaturized ultrasound equipment^1^. This innovation has led to the introduction of a concept of point-of-care ultrasound (POCUS), which enables non-specialist practitioners to conduct focused, brief ultrasound examinations at the bedside^2,3^. POCUS has gained traction in various domains, predominantly in emergency settings, encompassing cardiovascular applications.

Despite its versatility, accuracy remains a prevalent concern in POCUS^4,5^. Proficient ultrasound examination necessitates a specific level of training; however, not all healthcare professionals engaged in POCUS have undergone sufficient instruction. In the cardiovascular domain, one of the most important but skill-dependent parameters is left ventricular ejection fraction (LVEF). Guidelines recommend the use of LVEF in the decision-making process in various cardiovascular diseases^6,7^, whereas studies have shown that LVEF has significant inter-observer variability even among expertized sonographers^8^. Thus, the standardization of LVEF in POCUS has become an imperative issue to address.

Artificial intelligence (AI), specifically machine learning techniques including deep learning, has substantially enhanced the accuracy of computer vision in recent years, enabling the automatic processing of a wide range of information^9–13^. Medical imaging is no exception, with numerous publications documenting deep learning algorithms that automatically classify and analyze ultrasound images. Studies have shown that AI-based programs for automatic LVEF quantification are feasible^14–16^; however, the majority of these studies used high-end ultrasound equipment^17^. Emerging applications of AI-based automatic analysis on images obtained from handheld ultrasound devices on actual patients could contribute to addressing concerns about the accuracy of POCUS.

In light of these observations, we sought to investigate the real-world clinical feasibility of a novel deep learning-based automatic LVEF analysis program that is available with a handheld ultrasound device.

## Methods

### Patient enrollment

This study was a multicenter prospective observation including four centers in Japan (two image acquisition centers, one image analysis core laboratory, and one statistical analysis core laboratory). Patients who underwent clinically-indicated echocardiography in two hospitals were enrolled. After routine clinical echocardiography using high-end equipment (standard-echo), the same patient was scanned using a handheld device (KOSMOS, EchoNous Inc.) with an automatic LVEF analysis system (AI-POCUS). Inclusion criteria were (a) adult (20 years old or older) patients and (b) patients who can understand the study overview and give written informed consent. Exclusion criteria included (a) patients with congenital heart disease, (b) patients with a previous history of cardiac surgery, and (c) patients with arrhythmia during the examination.

The study protocols complied with the Declaration of Helsinki and were approved by the institutional review board of each hospital (Juntendo University Clinical Research Review Committee, Tokyo Bay Urayasu Ichikawa Medical Center Institutional Review, the Institutional Review Board of the Osaka City General Hospital, and Institutional Review Board of the University of Tokushima). Written informed consent was taken from all participants before joining the study.

### Data acquisition and analysis

Standard-echo was performed by a clinical sonographer in the two centers, recording apical two- and four-chamber views for at least three cardiac cycles. Subsequently, a cardiologist who was blinded to the results of standard-echo scanned the patients using the POCUS machine and acquired 5-s videos of apical two- and four-chamber views. Internally, the videos were analyzed to calculate LVEF by a deep learning-based program in the device. In this study, however, the cardiologists used a custom version of the device that does not display automatic LVEF results so that they can perform unbiased image acquisition. In other words, the cardiologists were blinded to the AI-POCUS LVEF and the endocardial borders that the algorithm depicted.

**Figure 1:**
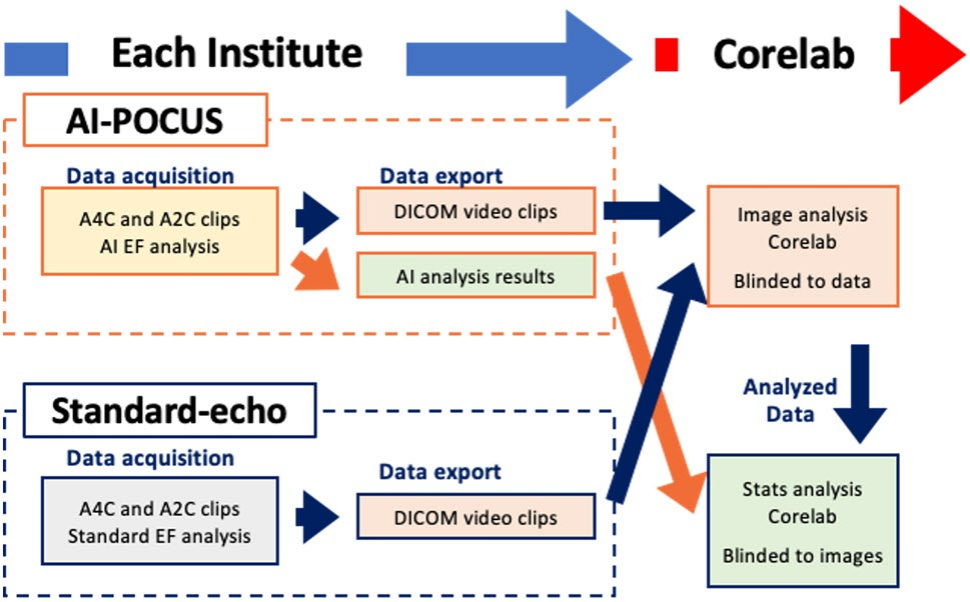
Study pipeline. Data acquisition was performed in each hospital both for standard-echo and for AI-POCUS. Acquired DICOM video clips of standard-echo were sent to the image-analysis core-laboratory, where all images were analyzed offline. The data from AI-POCUS (without going through image-analysis core-laboratory) and the results from image-analysis core-laboratory were sent to statistical-analysis core-laboratory.

The standard-echo images were transferred to the image analysis-core lab, where an expert cardiologist who was blinded to the AI-POCUS results analyzed LVEF for all standard-echo images with a manual biplane method of discs in accordance with published guidelines^6^. The images acquired using the POCUS machine were automatically analyzed at the time of scanning by the deep learning-based program, which had been developed by the company (EchoNous Inc.) using a completely different dataset. In this study, two different versions of the program were applied to the images in order to investigate the improvement of the software in the newer version. The following results present the results from the older version if not particularly mentioned because this version was commercially available at the time of drafting this manuscript in August 2023. These AI-POCUS results were transferred not to the image analysis-core lab, but to the statistical analysis-core lab, where a researcher compared the manual standard-echo data and the AI-POCUS data. This researcher was blinded to all echocardiographic images. Figure 1 shows the overall study pipeline.

Image quality was classified into three grades as follows; (a) good: all segments are visible throughout the cardiac cycle, (b) fair: one to two segments were poorly visible, and (c) poor: three or more segments were poorly visible in the six segments from apical views.

### Statistical analysis

Data are presented as mean ± standard deviation or medians [1st and 3rd interquartile ranges] for continuous variables as appropriate, and as frequencies (%) for categorical variables. Group differences were evaluated using Mann–Whitney U tests for continuous variables and the chi-square test or Fisher’s exact tests for categorical variables. Confidence intervals for sensitivities and specificities were calculated using exact Clopper–Pearson confidence intervals.

Consistency between the LVEF with standard-echo and that with AI-POCUS were assessed using intraclass correlation coefficients (ICC), and Bland–Altman plots were drawn to check the systematic bias between these two LVEF values. Limits of agreement were calculated as ± 1.96 standard deviations. LVEF was also categorized into reduced (< 50%) or preserved (≥ 50%) in accordance with clinical classification, and sensitivity, specificity, accuracy, and positive and negative predictive values of AI-POCUS were calculated with the standard-echo as clinical standard values. Subgroup analyses were performed for subtypes of patients based on the presence/absence of coronary artery disease, wall motion abnormality, and the institute of data acquisition. Using the Fisher r-to-z transformation, the significance of the difference between two correlation coefficients was assessed as the difference between two z values. All statistical analyses were performed with MedCalc version 20.218 (MedCalc Software Ltd, Ostend, Belgium) and R version 4.3.2 (The R Foundation, Vienna, Austria). In all analyses, a two-tailed p < 0.05 indicated statistical significance.

## Results

### Feasibility and accuracy of automated LVEF

Among a total of 200 enrolled patients, one patient was excluded because of a history of previous mitral valve surgery. Image quality assessed by the human reader was significantly better for the standard-echo images (good 197, fair 1, poor 1) than POCUS images (good 169, fair 22, poor 8; p = 0.001). These patients with poor image quality were excluded from the analysis. Additionally, eight cases were excluded because the images were rejected by AI-POCUS with the older software (version 2.0) due to insufficient quality. The number of rejections was significantly greater when using the latest version of the software (version 3.0, exclusion = 31, p < 0.001 vs. version 2.0), although the reasons for these rejections were not clear. Interestingly, these images judged as suboptimal by the AI-POCUS program were classified as good image quality by the human reader. Table 1 summarizes the patient characteristics of 182 patients whose images were analyzed by the software version 2.0. The mean age was 63.2 ± 14.9 years old, and LVEF by standard-echo was 47.8 ± 12.6 [ranges 14.9–70.7].

**Table 1:** Patient background.

	Values
Age, years	63.2 ± 14.9
Male, n (%)	137 (75.3%)
Height, cm	165 ± 9
Weight, kg	63.7 ± 14.4
BSA, m^2^	1.69 ± 0.21
BMI, kg/m^2^	23.3 ± 4.2
Underlying cardiac disease	
Coronary artery disease	74 (40.7%)
Dilated cardiomyopathy	25 (13.7%)
Hypertrophic cardiomyopathy	2 (1.1%)
Valvular heart disease	21 (6.6%)
Others/undetermined	42 (23.8%)
None	13 (7.1%)
Heart rate, /min	66.1 ± 14.4
LVEF (standard-echo) (%)	47.8 ± 12.6 [ranges 14.9–70.7]
LVEF (standard-echo) < 50%	85 (53.3%)
Regional wall motion abnormality	63 (34.6%)

*BSA* body surface area, *BMI* body mass index, *LVEF* left ventricular ejection fraction.

**Figure 2:**
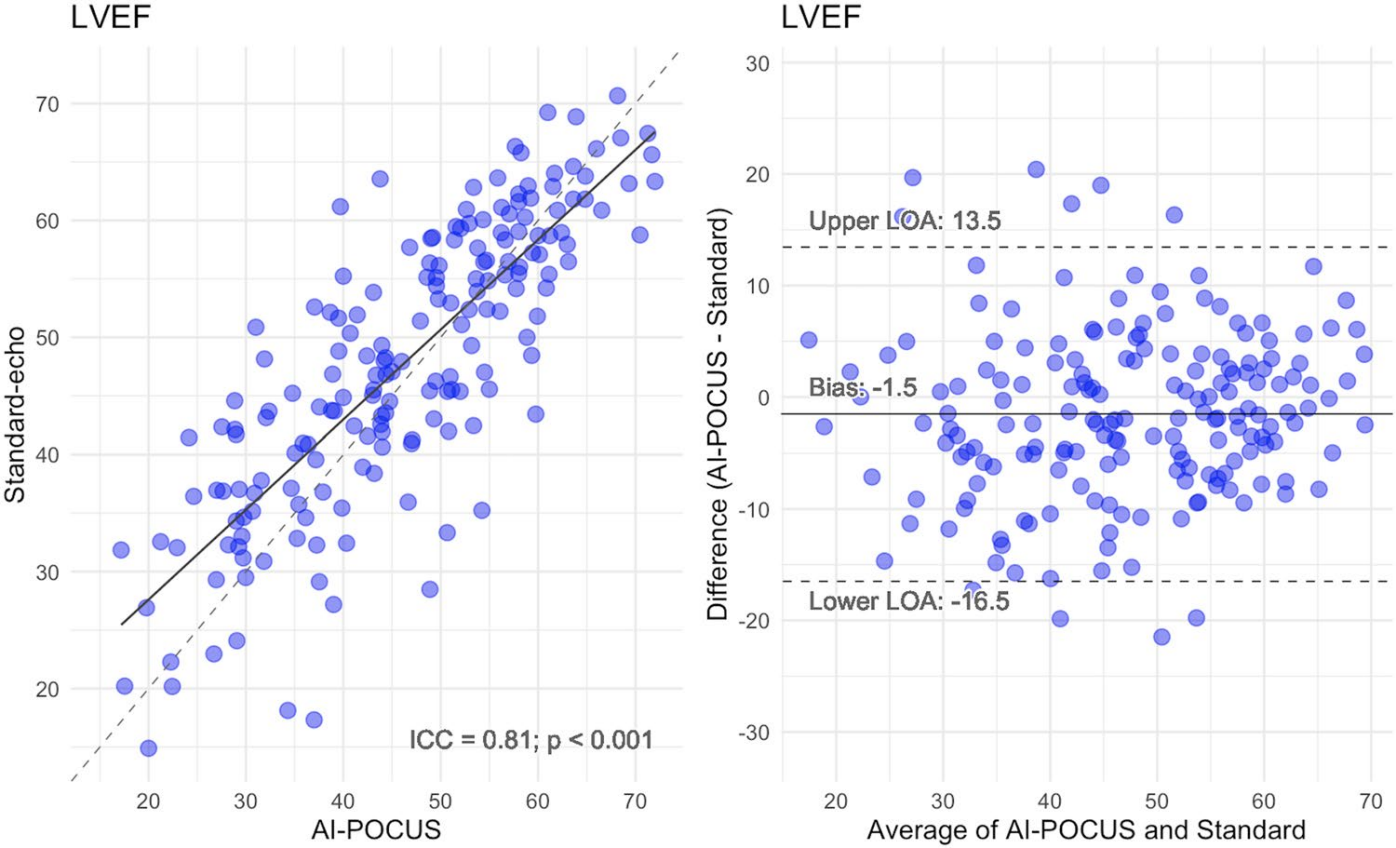
Correlation and bias in LVEF of AI-POCUS vs standard-echo. LVEF by AI-POCUS showed a good consistency with that by standard-echo (ICC = 0.81, p < 0.001) without systematic bias (mean bias − 0.2%, limits of agreement ± 15%).

**Figure 3:**
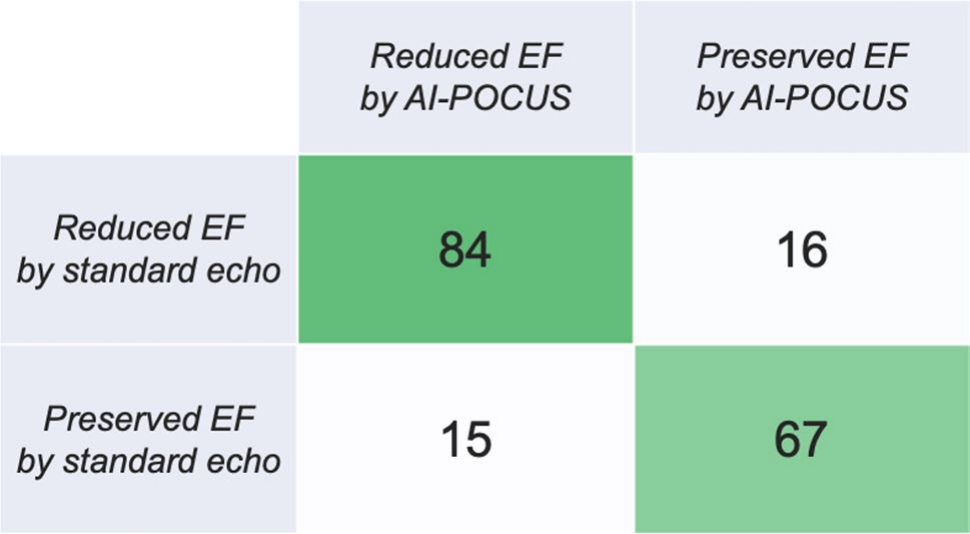
Accuracy of AI-POCUS to detect reduced LVEF. AI-POCUS detected reduced LVEF < 50% with a sensitivity of 85% (95% confidence interval 76%–91%) and specificity of 81% (71%–89%).

Figure 2 demonstrates the correlation between LVEF by standard-echo and that by AI-POCUS (panel A) and Bland–Altman plots (panel B). LVEF by AI-POCUS showed a good correlation with that by standard-echo (ICC = 0.81, p < 0.001) with minimal systematic bias (mean bias − 1.5%, limits of agreement ± 15.0%). As shown in the confusion matrix in Fig. 3, reduced LVEF < 50% was detected with a sensitivity of 85% (95% confidence interval 76%–91%) and specificity of 81% (71%–89%) by AI-POCUS.

### Subgroup of sites, body sizes, and LV wall motions

**Figure 4:**
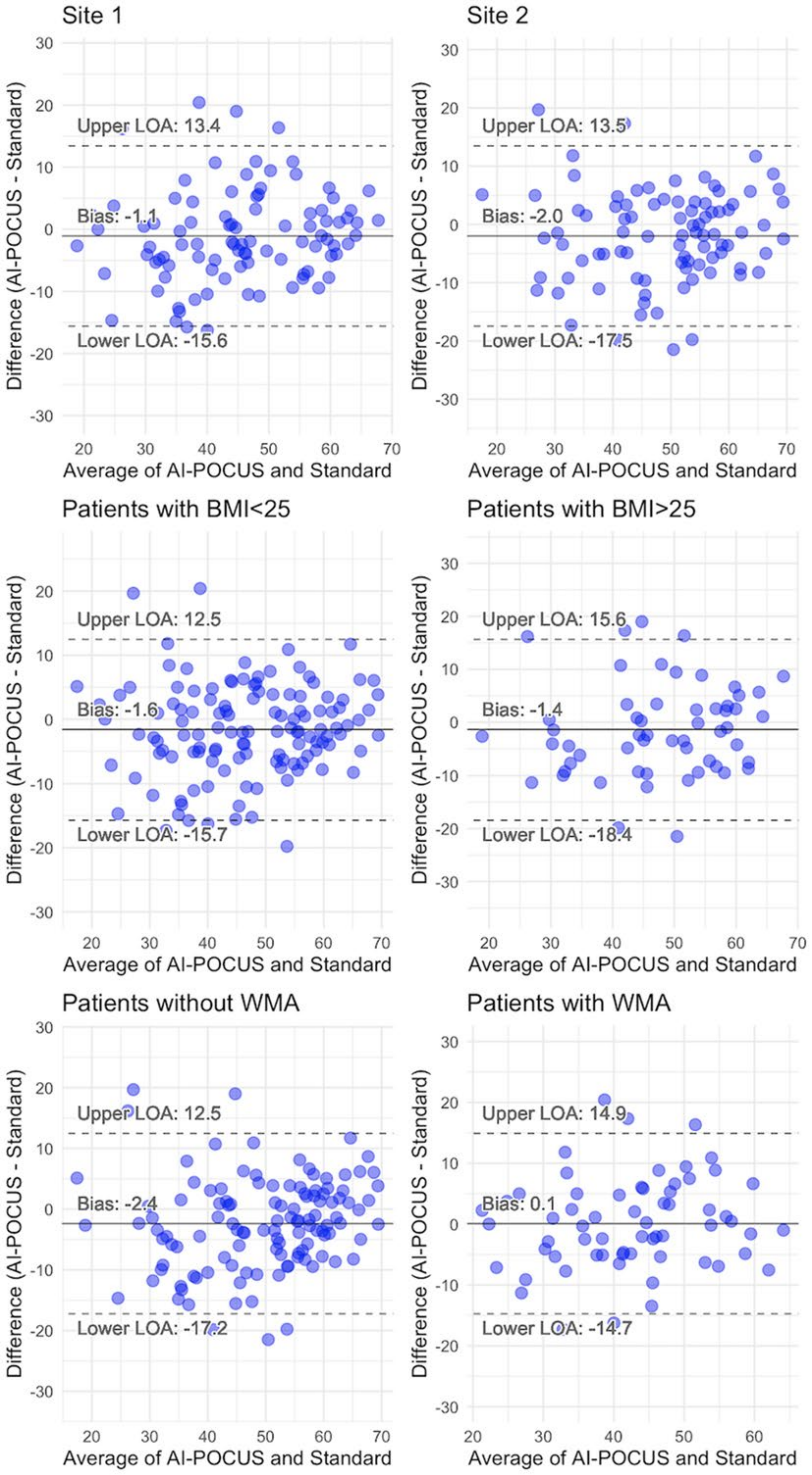
Correlations of LVEF in subgroups. Bland–Altman plots revealed that the bias and limits of agreement were not significantly different across the subgroups of sites (panel **A**, **B**), body mass index (panel **C**, **D**), and wall motion abnormalities (**E**, **F**). Differences in limits of agreement of LVEF were 2% to 3%, which are clinically acceptable.

The results of the subgroup analyses are summarized in Fig. 4. The bias and limits of agreement did not differ significantly across the subgroups of data acquisition sites, body mass index, or the presence or absence of regional wall motion abnormalities. The differences in bias and limits of agreement for LVEF were 2% to 3%, which are clinically acceptable. Importantly, although the images were acquired in two distinct sites (one university hospital and one public hospital), the accuracy of LVEF assessment was not different between the sites and systematic bias was not seen in either hospital (limits of agreement 13.4% to − 15.6% and 13.5% to − 17.5%). Similarly in the subgroups of body mass index and that of regional wall motion abnormality, no significant difference in the accuracy of LVEF was observed.

### LV volume quantification

**Figure 5:**
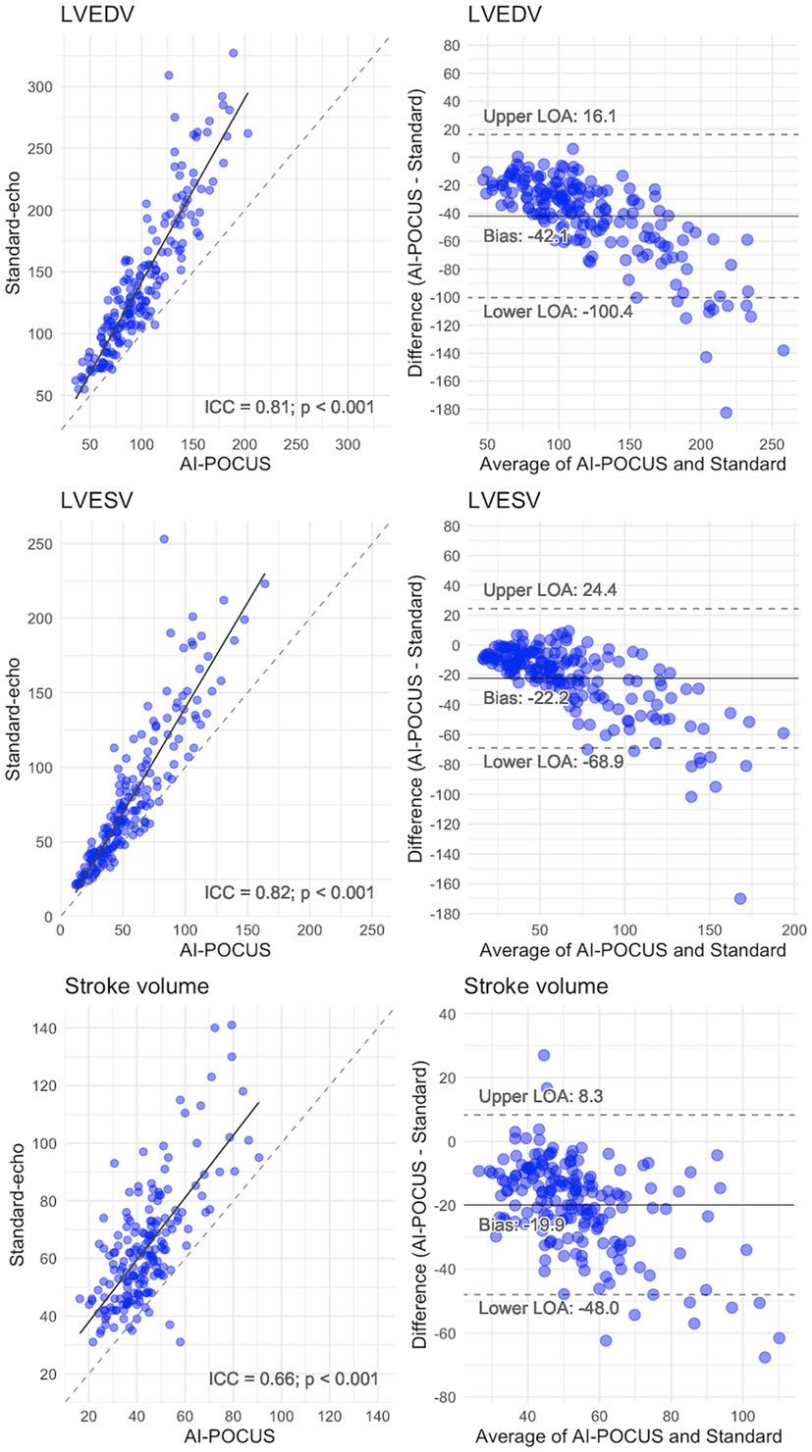
LV and stroke volumes. Scatterplots and Bland–Altman plots show the correlations and systematic bias of LV end-diastolic volume (panel **A**, **B**), end-systolic volume (panel **C**, **D**), and stroke volume (panel **E**, **F**).

Correlations and agreements of LV volumes and stroke volumes between standard-echo and that by AI-POCUS were shown in Fig. 5. Although consistencies were good in LV end-diastolic and end-systolic volumes (ICC = 0.81 and 0.82, respectively, p < 0.001 for both), AI-POCUS tended to underestimate volumes especially when they are greater (bias of 42.1 mL for LV end-diastolic volume). As shown in the following section and in the Supplementary materials, however, these trends of underestimation became smaller when the latest version of the software, maintaining similar consistencies.

### Influence of software version

The same analyses using the latest version of the software (version 3.0) are summarized in the supplementary materials (Supplementary Figs. 1 to 4). Detailed differences in the development process are industrial secrets, however, the basic principle of the upgrade was to increase the size of the dataset for training deep learning models and improving the deep learning architecture. Overall, the results by the latest software had slightly better consistencies with standard-echo. However, the newer version of the software accepted a narrower range of images than the older version, as mentioned above. Notably, the trends of underestimation of the LV volumes in larger LVs were clearly smaller in the newer version compared with the older version (Fig. 5 and Supplementary Fig. 4).

## Discussion

In this real-world validation study, we have shown that (1) this deep learning-based AI-POCUS program that automatically quantifies LVEF was applicable in the majority of clinical images (91.5% of the real-world examinations); (2) LVEF by AI-POCUS showed a good concordance with standard-echo (ICC = 0.81, limits of agreement ± 15.0%) regardless of the image acquisition sites and other subgroups; (3) AI-POCUS underestimates LV volumes when the volumes are greater, although the degree of underestimation could be mitigated by updating the version of the software.

POCUS using handheld ultrasound devices has rapidly been spread to various medical situations including not only hospitals but also small clinics and pre-hospital medical scenes^18^. However, the interpretation of ultrasound images requires certain training and background medical knowledge. POCUS outside hospitals tends to be performed by less experienced medical staff, including non-physicians such as nurses and emergency team members. The image qualities of handheld ultrasound devices are generally inferior to those of high-end ultrasound machines which offer high-resolution supreme image quality, advanced features, and a broader range of transducer options^19^. These differences in image qualities also impact the applicability of automated image analysis programs. Although many commercial high-end machines have automated quantification programs for cardiac parameters including LVEF, very limited handheld ultrasound devices have such programs.

The device used in the present study was a handheld ultrasound device with AI-based programs. This device (KOSMOS, EchoNous Inc) offers AI-based programs for automated LVEF measurement despite its handheld size. The image quality of this device was not as comparable to a high-end machine as shown in our results, however, the AI program on this device was able to analyze such images and return a similar result as a human expert reader’s measurement of LVEF using a high-end machine. Advanced algorithms enabled by recent advancements in deep learning, like the one employed in our AI-POCUS application, are capable of compensating for some of the inherent limitations of handheld devices by optimizing image processing and analysis^20,21^. As a result, our study results might demonstrate that the gap between high-end ultrasound machines and handheld devices in terms of diagnostic performance has narrowed, allowing for more accurate and reliable LVEF quantification even with a handheld device.

A recent study by Papadopoulou and colleagues also tested the ability of this same AI-POCUS automatic LVEF program^22^. The results were mostly consistent with our results. However, our study provides additional intriguing findings, including the reduced accuracy of AI-based programs in larger LVs and the improvement in accuracy with the newer version of the software, which was trained on a larger dataset. In general, when a part of the data is scarce, a machine learning model often fails to learn it properly, resulting in decreased performance and accuracy of the model^23,24^. Details of the development process of the present program are confidential, however, the newer version of the model was trained with a greater number of patients with enlarged LV according to the company. Thus, the present results showing improvement in the performance of LV size analysis with the newer version of the software further emphasize the importance of including larger, highly heterogeneous datasets for training AI-based programs.

Our results have clinical implications, as the AI-POCUS application offers a reliable, convenient, and non-invasive tool for the rapid assessment of LVEF in diverse clinical settings. The ability to accurately quantify LVEF with a handheld ultrasound device can enhance the efficiency and diagnostic capabilities of healthcare professionals, particularly in situations where access to a full-scale echocardiography laboratory may not be feasible. By reducing the need for manual calculations, the AI-POCUS application can facilitate quicker and more informed decision-making in the management of patients with cardiovascular disorders. However, clinicians should be aware of the limitations of the present AI-POCUS application in larger LV volumes and consider corroborating the findings with standard echocardiography when necessary.

## Limitations

This study is best understood in the context of several limitations. First, despite a multi-center observation, the number of hospitals where the images were acquired was only two, both of which are large, university-level hospitals. Further studies including a wider range of medical facilities should confirm the present results. Another important limitation is that all AI-POCUS was performed by expert echocardiographers who were capable of acquiring clear apical 4-chamber views. For novice observers, additional technologies such as AI-acquisition guidance or tele-ultrasound solutions might be necessary to help acquire appropriate images^5,25,26^. Next, since the AI-POCUS was a commercial program that had been developed by the company, details of the model architecture and dataset with which the program was developed were unknown. This study did not include all patients who were referred to echocardiography in our hospitals, and we might exclude patients whose images were obviously poor. However, in clinical practice, manual LVEF measurement is also impossible for such patients and thus it should be acknowledged as the limitation of echocardiography itself, not particularly in this program. Finally, we used LVEF measured by standard-echo as clinical standard values; however, it is well known that LVEF by 2D methods has significant variability and a non-negligible difference from the gold standard measurements obtained by magnetic resonance imaging. Thus, it should be acknowledged that there is a risk that the label itself (reduced or preserved LVEF) might differ when using the gold standard.

## Conclusions

In this real-world multicenter study performed by expert cardiologists, the AI-POCUS was feasible in the assessment of LVEF. Careful attention might be necessary when applying the program to larger LV. These results should be acknowledged by clinicians as well as researchers who develop future AI-POCUS.

### Supplementary Information

Supplementary Figures.

## Data Availability

The datasets used and/or analyzed during the current study available from the corresponding author on reasonable request.

## Abbreviations

AI: Artificial intelligence
POCUS: Point-of-care ultrasound
LV: Left ventricle
LVEF: Left ventricular ejection fraction
