# Supplementary material for: Multicenter validation study for automated left ventricular ejection fraction assessment using a handheld ultrasound with artificial intelligence
Source: Sci Rep. 2024 Jul 4;14:15359. doi: 10.1038/s41598-024-65557-5 (PMC11224326; doi:10.1038/s41598-024-65557-5)
Supplement: Supplementary file 1 — Supplementary Figures. [file 41598_2024_65557_MOESM1_ESM.docx]

# Supplemental Material: Multicenter validation study for automated left ventricular ejection fraction assessment using a handheld ultrasound with artificial intelligence

## Supplemental Figure 1. LVEF of AI-POCUS with the newer version of the software vs standard-echo

LVEF by AI-POCUS with the newer version of the software showed an excellent correlation with that by standard-echo (ICC = 0.83, p<0.001) without systematic bias (mean bias -0.2%, limits of agreement ±13.7%).


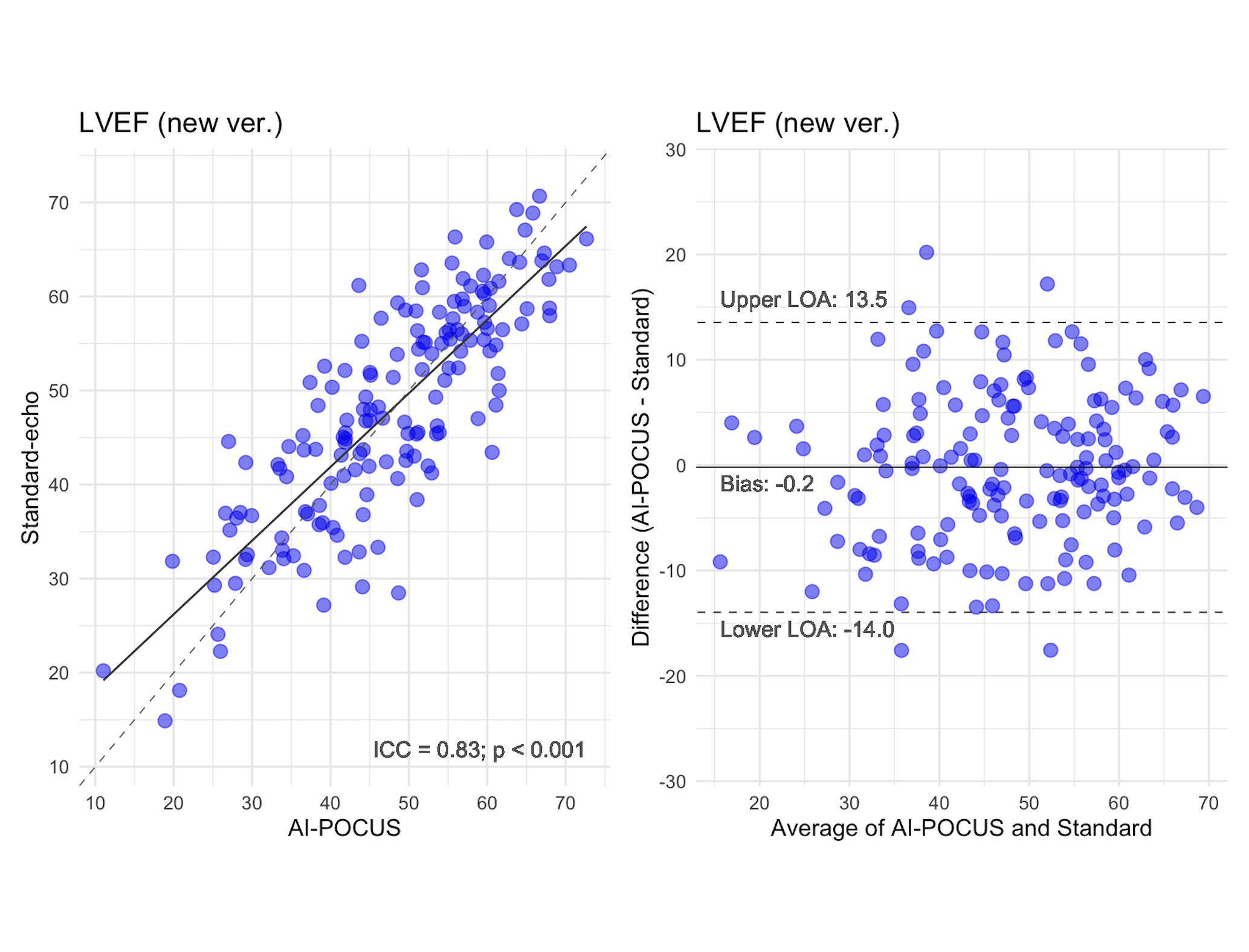


## Supplemental Figure 2. AI-POCUS with the newer version of the software to detect reduced LVEF

AI-POCUS with the newer version of the software detected reduced LVEF < 50% with a sensitivity of 85% (95% confidence interval 75.3% – 91.6%) and specificity of 84% (73.4% – 91.3%).


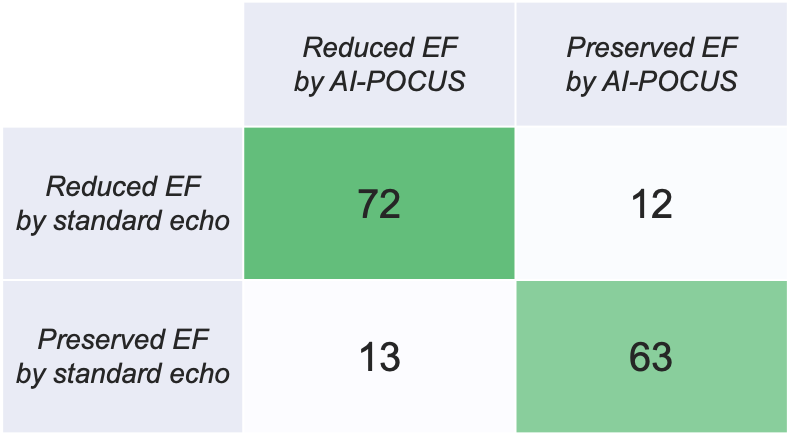


## Supplemental Figure 3. LVEF with the newer version of the software in subgroups

Bland-Altman plots across the subgroups of sites (panel A and B), body mass index (panel C and D), and wall motion abnormalities (E and F). Findings were mostly similar to those of the older version of the software.


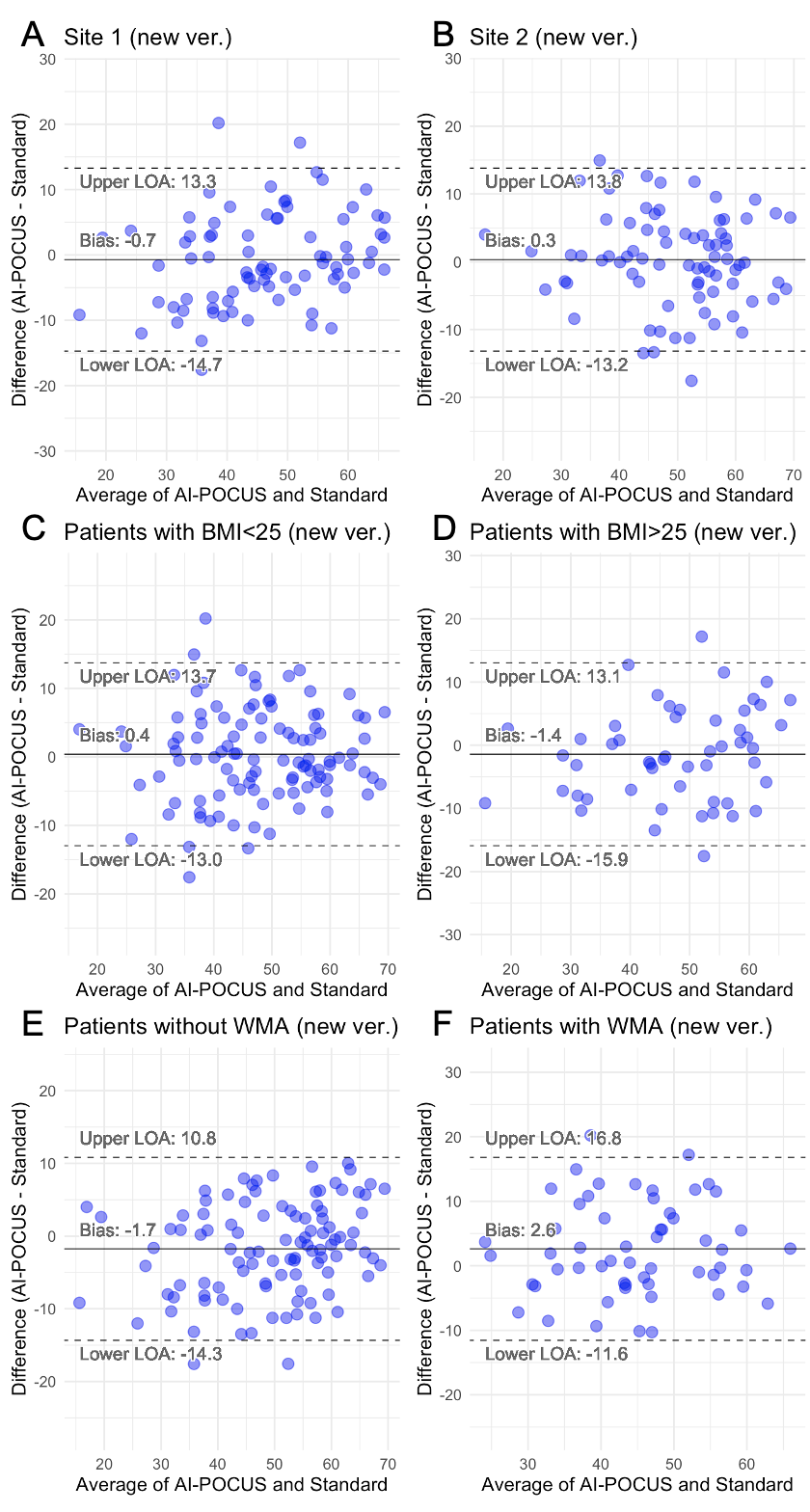


## Supplemental Figure 4. LV and stroke volumes with the newer version of the software

Scatterplots and Bland-Altman plots of LV end-diastolic volume (panel A and B), end-systolic volume (panel C and D), and stroke volume (panel E and F). The trends of underestimation seen in the older version became smaller with the latest version of the software.
